# Blood Pressure Non-Dipping and Obstructive Sleep Apnea Syndrome: A Meta-Analysis

**DOI:** 10.3390/jcm8091367

**Published:** 2019-09-02

**Authors:** Cesare Cuspidi, Marijana Tadic, Carla Sala, Elisa Gherbesi, Guido Grassi, Giuseppe Mancia

**Affiliations:** 1 Department of Medicine and Surgery, University of Milano-Bicocca, 20036 Meda, Italy; 2 Istituto Auxologico Italiano, 20122 Milano, Italy; 3 Department of Internal Medicine and Cardiology, Charité–Universitätsmedizin Berlin, Augustenburgerplatz 1, 13353 Berlin, Germany; 4 Department of Clinical Sciences and Community Health, University of Milano and Fondazione Ospedale Maggiore IRCCS Policlinico di Milano, 20122 Milano, Italy

**Keywords:** obstructive sleep apnea, non-dipping pattern, meta-analysis

## Abstract

Aim: We examined the reduced blood pressure (BP) nocturnal fall in patients with obstructive sleep apnea (OSA) by a meta-analysis including studies that provided data on prevalence rates of non-dipping (ND) pattern during 24-h ambulatory blood pressure monitoring (ABPM). Design: The PubMed, OVID-MEDLINE, and Cochrane CENTRAL literature databases were searched for appropriate articles without temporal restriction up to April 2019 through focused and sensitive search methods. Studies were identified by crossing the search terms as follows: “obstructive sleep apnea”, “sleep quality”, “non dipping”, “reduced nocturnal BP fall”, “circadian BP variation”, “night-time BP”, and “ambulatory blood pressure monitoring”. Results: Meta-analysis included 1562 patients with OSA from different clinical settings and 957 non-OSA controls from 14 studies. ND pattern prevalence in patients with OSA widely varied among studies (36.0–90.0%). This was also the case for non-OSA controls (33.0% to 69.0%). Overall, the ND pattern, assessed as an event rate in the pooled OSA population, was 59.1% (confidence interval (CI): 52.0–65.0%). Meta-analysis of the seven studies comparing the prevalence of ND pattern in participants with OSA and controls showed that OSA entails a significantly increased risk of ND (Odds ratio (OR) = 1.47, CI: 1.07–1.89, *p* < 0.01). After the exclusion of patients with mild OSA, OR increased to 1.67 (CI: 1.21–2.28, *p* < 0.001). Conclusions: The present meta-analysis, extending previous information on the relationship between OSA and impaired BP dipping, based on single studies, suggests that this condition increases by approximately 1.5 times the likelihood of ND, which is a pattern associated with a greater cardiovascular risk than normal BP dipping.

## 1. Introduction

Ambulatory blood pressure monitoring (ABPM) provides the unique opportunity to assess circadian blood pressure (BP) variability [1]. A large amount of evidence has demonstrated that night-time systolic and diastolic BP values are 10–20% lower than corresponding day-time values in the large majority of normotensive healthy subjects and uncomplicated hypertensive patients [2]. The nocturnal BP fall is closely linked to the physiological reduction of the sympathetic nervous system during the night-time period, which results in a pronounced decrease in cardiac output, arterial resistances, and heart rate [3].

The extent of day/night BP variations in population-based samples and, in general, hypertensive cohorts has been related to several factors such as age, ethnicity, intensity of diurnal physical activity, job stress, smoking habits, quality of sleep, seasonal influence, and co-morbidities [4,5,6].

A reduced BP fall at night (the defined non-dipping, defined as a <10% BP reduction of night-time vs. daytime BP or night-time/day-time BP ratio ≥0.90) has been reported to be highly frequent in several morbid conditions, including obstructive sleep-apnea syndrome (OSA) [7,8,9].

OSA, which is often associated with hypertension, obesity, diabetes, and metabolic syndrome, is a highly prevalent chronic condition in the general population, characterized by a marked stimulation of the sympathetic activity induced by recurrent nocturnal desaturation episodes [10].

Since increased sympathetic activity has been proposed as one of the major mechanisms responsible of alterations in BP and circadian BP rhythm (i.e., non-dipping and reverse pattern), numerous studies have targeted the association of OSA with BP disorders [11,12]. Regarding the relationship of OSA with the non-dipping (ND) pattern, available evidence is so far limited to relatively few investigations, based on small-scale studies, conducted in different clinical settings and, more importantly, with inconsistent conclusions [13].

Taking all these aspects into account, we, therefore, decided to make a systematic meta-analysis of the studies that addressed this specific topic with the primary aim to expand available evidence in this research field.

## 2. Materials and Methods

### 2.1. Search Strategy and Study Selection

The present study was carried-out following the Preferred Reporting Items for Systematic reviews and Meta-Analyses (PRISMA) guidelines [14]. Pertinent literature was examined to detect all papers providing findings on the association of OSA with the ND pattern, as assessed by ABPM.

The PubMed, OVID-MEDLINE, and Cochrane CENTRAL databases were systematically analyzed for English-language research articles without time restriction until April 2019.

Studies were detected by using the following terms: “obstructive sleep apnea”, “sleep quality”, “non dipping”, “reduced nocturnal BP fall”, “reverse dipping”, “circadian BP variation”, “night-time BP”, and “ambulatory blood pressure monitoring”. Checks of the reference lists of selected papers integrated the electronic search. Reviews, editorials, case reports, and letters were excluded from analyses, but were examined for potential additional references. Two authors (CC and EG) assessed retrieved abstracts and full text of these studies to establish eligibility, according to the inclusion criteria mentioned below. A third reviewer (CS) resolved disagreements on study judgments. Data extraction were performed by one reviewer (CC) and independently checked by another reviewer (CS).

Inclusion criteria were: (1) English articles published in peer-reviewed journals, (2) studies providing information on the prevalence of the ND pattern in OSA individuals, and (3) minimum set of data including age, gender, Body surface area (BSA), or body mass index (BMI). Specific exclusion criteria were: (1) studies with less than 10 patients with OSA, and (2) studies conducted in children and adolescents (age <18 years).

**Figure 1:**
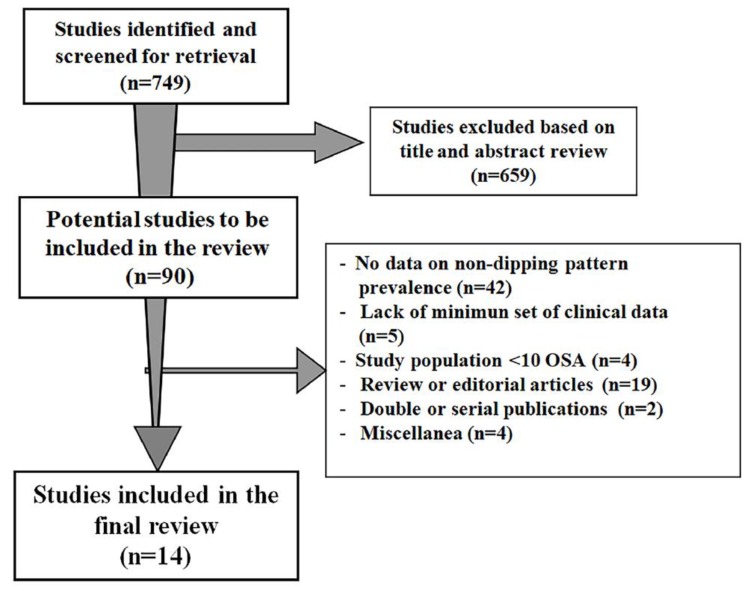
Schematic flow-chart for the selection of studies.

The first literature screening identified a total of 159 papers. After the initial search of titles and abstracts, 659 papers were excluded and 90 were reviewed. Of these, 14 studies [15,16,17,18,19,20,21,22,23,24,25,26,27,28] fulfilled the inclusion criteria and comprised sufficient data to be enclosed in the present review (Figure 1).

### 2.2. Definitions

Ambulatory blood pressure measurements and definition of the non-dipping status.

ABPM was performed by using validated devices in all studies. Spacelabs 90207 (Spacelabs, Redmond, WA, USA) was the most frequently employed monitoring equipment (7 studies), which is followed by FM 800 or FB 250 (Fukuda, Denshy Ltd., Tokio, Japan) (3 studies). Instruments were set to take BP readings at different time intervals (day-time:15–30 min and night-time: 20–60 min) (Supplementary Table S1).

Patients were defined as non-dippers (data provided by 12 out of 14 studies) when mean night-time BP (5 studies), systolic/diastolic BP (4 studies), and systolic BP (3 studies) decreased by ≤10% compared to day-time values.

The night-time period was defined according to five different criteria (data from 13 studies). Day–time and night-time periods were classified according to an individual’s diary in six studies [19,23,24,26,27,28]. Fixed time intervals were used to define the night-time period in the remaining studies: 10.00 PM–6.00 AM (four studies) [15,17,21,25], 10.00 PM–7.00 AM [18], 11.00 PM–6.00 AM [20], and 12.00 PM–6.00 AM [16].

### 2.3. Obstructive Sleep Apnea Syndrome Definition

In the selected studies, respiratory events were scored according to the recommendations of major guidelines as follows: an apneic event was defined as ≥90% decrease in airflow from the baseline value for ≥10 s. Hypopnea was variously classified as a 30–50% decrease in airflow lasting at least 10 s that may or may not be associated with arousals or 3–4% oxy-hemoglobin desaturation. OSA was defined according to the apnea/hypo-apnea index (AHI) cut-off of ≥15 events/h in eight studies, ≥5 events/h in six studies, and ≥30 events/h in one study. Diagnosis of OSA was made out-of-hospital by unattended home sleep recordings via polysomnography in four studies [15,20,26,28] and by polygraphy in three studies [21,24,27], respectively, and in the hospital setting (sleep laboratory) in the other seven studies [16,17,18,19,22,23,25] by using different commercial devices.

### 2.4. Statistical Analysis

The aim of the review was to assess the prevalence of ND pattern in the OSA setting, according to the criteria provided by the studies. In order to calculate average ND pattern prevalence in the entire population, we performed a meta-analysis of data provided by the 14 selected studies and considered ND occurrence as an event rate.

Clinical features retrieved by selected studies are expressed as absolute numbers, mean± standard deviation (SD), or inter-quartile range.

Odds ratios (ORs) with 95% confidence intervals (CIs) were calculated in order to evaluate the statistical difference of the ND pattern frequency between OSA and controls (data provided by seven studies).

A pooled analysis of the previously mentioned variables was carried out using fixed or random effects models by Comprehensive Meta-Analysis Version 2, Biostat, Englewood, NJ. The limit of statistical significance was set at *p* < 0.05.

Heterogeneity was assessed by using I-square, Q, and tau-square values. The random effect model was applied when heterogeneity across studies was high (*I*^2^ > 75). Relevant publication bias was evaluated by using the funnel plot method, according to the trim and fill test. Among observed and adjusted values, their lower and upper limits have been calculated.

## 3. Results

Overall, 2519 participants (1562 with OSA and 957 controls) were enrolled in 14 studies (sample size ranged from 44 to 703), performed in three different geographical areas (Asia = 7, South America = 3, North America = 2, Europe = 2). Notably, 1672 subjects (66.4%) were examined in Asia, 342 subjects (13.5%) in North America, 329 subjects (13.1%) in South America, and 177 subjects (7.0%) in Europe.

Table 1 reports the main features of selected studies comprising year of publication, OSA sample size, presence of controls, prevalence of men, mean age, body mass index (BMI), night-time BP, clinical setting, and definition of OSA. Six out of 14 studies included untreated or treated hypertensive patients, three studies included normotensive individuals from different settings (obese, elderly, and apparently healthy subjects), and other studies investigated mixed normotensive/hypertensive samples, members of the general population, patients with cardiovascular disease, or focused on severe OSA. Only seven studies included non-OSA individuals as a control group.

### 3.1. Clinical Characteristics of Patients with OSA

Average age range was 44 to 77 years [18,24] and 84% of participants were men. Mean BMI varied from 24.0 ± 3.0 kg/m^2^ (20) to 34.0 ± 6.0 kg/m^2^ [21] (data provided by 13 studies). Average night-time systolic BP ranged from 115 ± 8 mmHg [24] to 132 ± 16 mmHg [18] and diastolic BP from 70 ± 8 mmHg [24] to 81 ± 10 mmHg [28] (data from nine studies).

### 3.2. Prevalence of the ND Pattern

**Figure 2:**
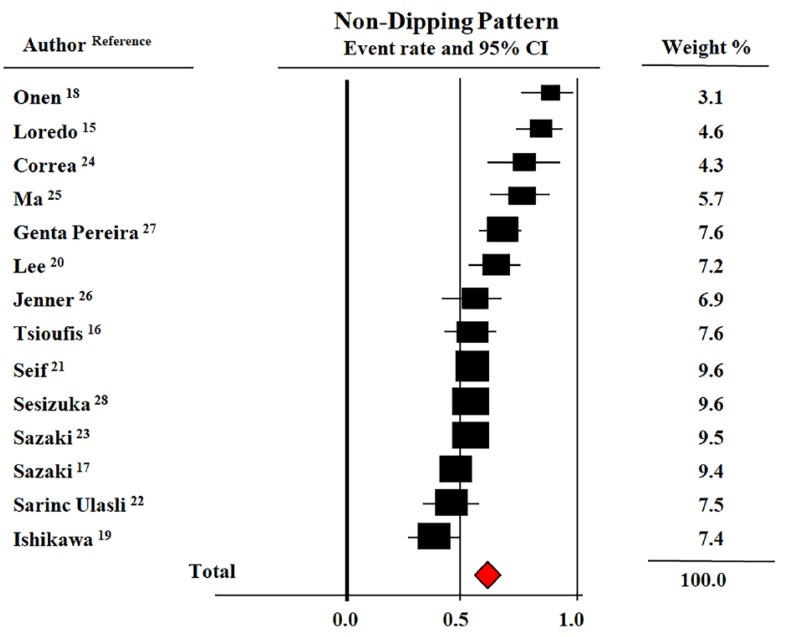
Forest plot of prevalence rates of a non-dipping pattern during 24-h blood pressure monitoring in patients with obstructive sleep apnea (OSA) [15,16,17,18,19,20,21,22,23,24,25,26,27,28]. Data from 14 studies and 1562 participants. Random model (*I*^2^ = 79%).

ND pattern prevalence in patients with OSA consistently varied among studies (36.0–90.0%). Corresponding figures in non-OSA controls ranged from 33% to 69%. Figure 2 shows the Forest plot of prevalence rates of the ND pattern in patients with OSA. The overall prevalence of this nocturnal BP phenotype, assessed as an event rate, in the pooled population was 59.1% (CI: 52.0–65.0%). An additional, separate analysis of studies that included only patients with mild OSA [16,17,22,23,25] showed a significant lower prevalence of the ND pattern in this subset (46%, CI: 46–65%) as compared to the whole pooled population.

**Figure 3:**
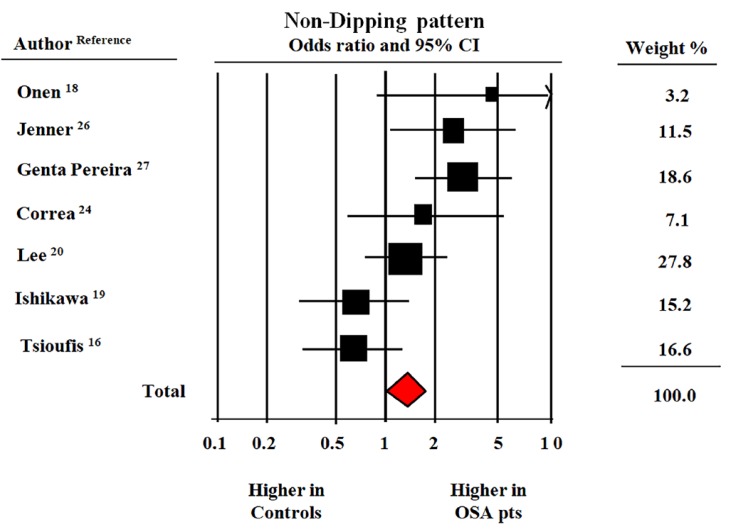
Odds ratio of the non-dipping pattern during 24-h blood pressure monitoring in patients with obstructive sleep apnea vs. without obstructive sleep apnea (OSA) [16,18,19,20,24,26,27]. Data from seven studies and 1330 participants with and without OSA. Fixed model (*I*^2^ = 56%).

The meta-analysis of the seven studies that provided data on the prevalence rates of the ND pattern in patients with OSA and in their non-OSA counterparts suggested that the former group had a significantly increased risk of a reduced fall in night-time BP (OR = 1.47, CI: 1.07–1.89, *p* < 0.01) (Figure 3).

**Figure 4:**
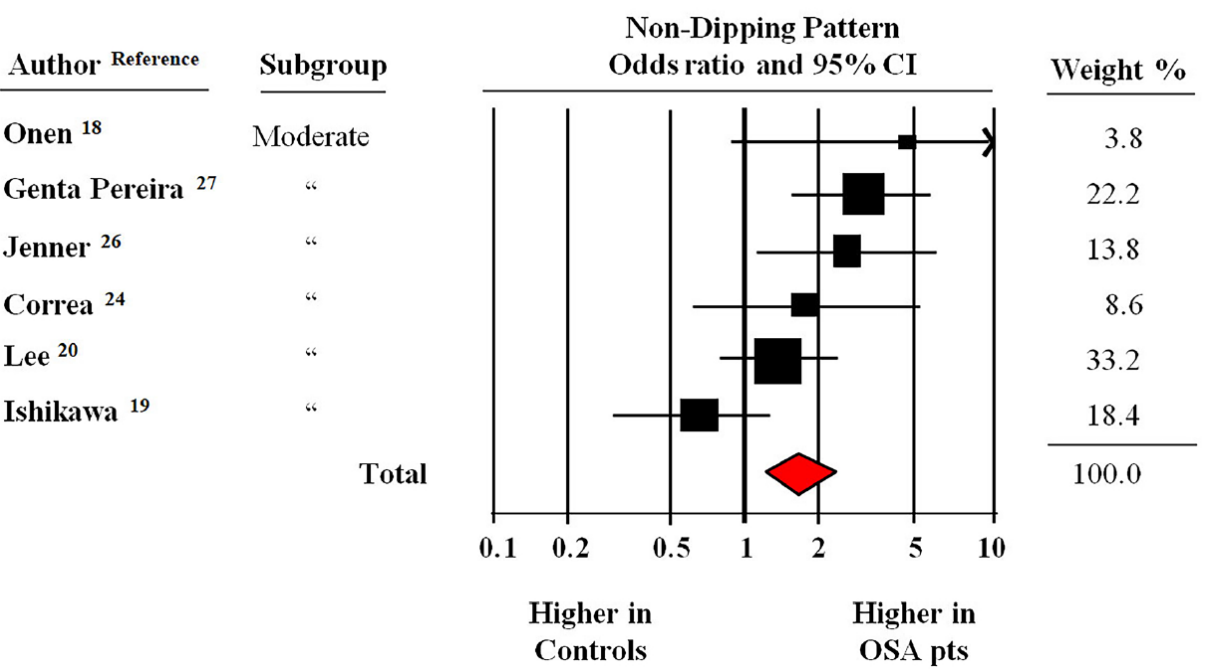
Odds ratio of non-dipping pattern during 24-h blood pressure monitoring in patients with obstructive sleep apnea (OSA) of moderate to a severe degree compared to controls [18,19,20,24,26,27]. Data from six studies and 1198 participants with and without OSA. Fixed model (*I*^2^ = 59).

Figure 4 reports the findings of the meta-analysis of six studies that excluded patients with AHI <15 events/hour (i.e., mild OSA). In this pooled population with a moderate to severe OSA, the likelihood to have an ND pattern was 1.67 times higher (CI: 1.21–2.28, *p* < 0.001) than the controls.

**Figure 5:**
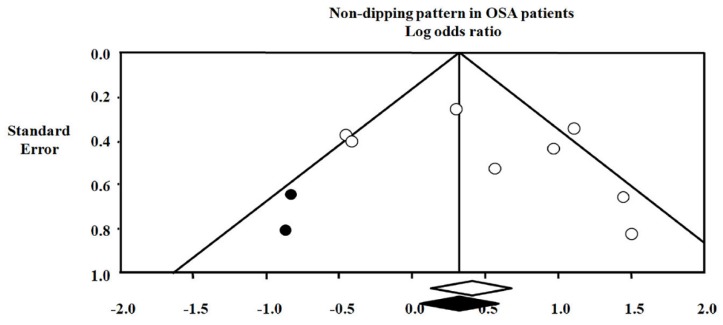
Publication bias of the non-dipping pattern in obstructive sleep apnea OSA patients: Observed odds ratio 1.50 (1.14–1.98). Adjusted odds ratio 1.37 (1.05–1.79). White symbols refer to observed data. Black symbols refer to adjusted data.

A funnel plot ruled out the presence of significant publication bias of studies by assessing the prevalence of the ND pattern in individuals with and without OSA (Figure 5). Lastly, the adjustment for publication bias did not abolish the difference in the ND pattern prevalence between OSA and controls.

## 4. Discussion

Our meta-analysis of 14 studies published in the last two decades adds a new piece of information on the relationship between OSA and the ND pattern in a pooled population from different settings totaling 2519 normotensive, untreated, and treated hypertensive individuals without and with mild (i.e., AHI ≥ 5 events/hour, five studies), moderate to severe (i.e., AHI ≥ 15 events/hour, eight studies) and severe (i.e., AHI ≥ 30 events/hour, one study) OSA.

We found that the prevalence rates of the ND pattern, as assessed by a single ABPM recording, in patients with OSA and controls widely varied among the selected studies (36.0–90.0% and 33.0% to 69.0%, respectively). This depends on the differences in demographic/clinical characteristics, definitions of OSA and ND phenotypes, as well as the modality of performing ABPM and polysomnography (i.e., home or hospital).

More importantly, we have been able to show that the ND pattern is present in most patients with OSA, with a pooled prevalence of 59.0%. Further vital information from our meta-analysis concerns the estimate of the magnitude of risk of reduced nocturnal BP dip associated with OSA. The probability of having an ND pattern in patients with OSA was 46% higher and 67% higher than controls, depending on whether subjects with mild OSA were included or excluded, respectively. These results suggest that this risk is linked to the OSA severity. Lastly, prevalence rates of ND as well as ORs entailed by OSA based on pooled data from the selected studies were un-affected by publication bias or a single effect study.

Before further commenting on the main findings and limitations of our study, some general aspects related to the prevalence of OSA and ND, as well as the mechanisms that determine alterations in circadian BP rhythm in the OSA setting, deserve to be briefly discussed.

Recent epidemiological studies have shown a rapid growth in the prevalence of sleep disordered breathing. Moderate to severe OSA, as defined by an apnea–hypopnea index (AHI) > 15, has been recently reported in up to one half of men and a quarter of women belonging to the general population [29]. At the same time, although obesity levels have risen dramatically, epidemic obesity does not fully explain the increasing burden of OSA. This is because the measurement techniques and diagnostic scoring criteria have also changed markedly over the same period. Cross sectional studies have reported independent associations between severity of OSA and hypertension after adjusting for confounders. Furthermore, a close relationship between OSA and BP disorders has been confirmed by observational and interventional studies in which OSA was found to be a major predictor of incident hypertension and an ND pattern [30].

In the same period, the widespread use of ABPM has documented that a reduced or even absent night-time BP occurs in a remarkable fraction of hypertensive cohorts (20–40%), independently from several variables such as ethnicity, age, and gender [31]. It is also noted that, even at the community level, the ND pattern is not rare. A pioneering contribution regarding ambulatory BP patterns in the general population comes from the Pressioni Arteriose Monitorate E Loro Associazioni (PAMELA) study, which is an observational survey carried out in 2051 Italian individuals, randomly selected from the residents in Monza (Milan, Italy) [32]. In the PAMELA population, ND prevalence varied from 15% to 20% depending on whether this nocturnal BP pattern was identified by systolic or diastolic BP nocturnal drop or both. Taken together, these data indicate that both OSA and non-dipping patterns are frequent conditions in the community.

There is a general consensus that the physiological nocturnal BP drop is largely due to a decrease in the sympathetic tone, which leads to a pronounced fall in both cardiac output and peripheral artery resistances. A large amount of reports has shown that plasma norepinephrine and epinephrine levels exhibit circadian variations with a nadir during night-time sleep [3].

Studies based on direct recording of muscle sympathetic nerve activity via microneurography have suggested that day-night BP difference is inversely related to sympathetic nerve traffic (i.e., the greater the sympathetic awake tone, the lesser the magnitude of nocturnal hypotension) [33]. There is also evidence of a link between the non-dipping pattern to an altered renal capacity to excrete sodium during the day. It has been shown that nocturnal BP throughout the pressure-natriuresis mechanism may lead to an increased sodium excretion, which determines a preservation of the sodium balance [11].

As for the relation between OSA and abnormal nocturnal BP dipping (i.e., ND and reverse dipping), the mechanisms by which this condition promote the presence of ND include recurrent hypoxaemia and hypercapnia, cortical microarousals, raised oxidative stress, and fragmented sleep, in which the synergistic effects lead to transient elevations of BP, likely via sympathetic activation. Intermittent hypoxia has been considered one of the main factors involved in the development of hypertension among patients with OSA [34]. In addition, emerging evidence suggests that sleep fragmentation is a major trigger factor for elevated BP in fragmented sleep and repeated arousals in patients with periodic leg movements have also been reported to be associated with hypertension.

It is reasonable to think, however, that, in addition to these unfavorable mechanisms, individual conditions can attenuate or counteract the pressor effects of OSA. This hypothesis is in line with the results of our meta-analysis. Despite that the majority of the pooled population with OSA exhibited an ND pattern, a normal circadian BP rhythm was found in a variable, non-marginal fraction of patients with OSA enrolled by single selected studies.

Some brief additional comments on the clinical features of the studies included in our meta-analysis may be useful. First, as expected, in the vast majority of the studies, OSA was associated with being overweight and obese. Second, average night-time systolic and/or diastolic BP values were equal or higher than 120/70 mmHg in all studies but one that provided this type of information, which suggests that nocturnal hypertension is a common feature in patients with OSA. Third, severe OSA was addressed by one specific study, and the prevalence of ND in this cohort was no different from that reported in other studies including less severe patients, which makes a conclusion on this topic unfeasible. Lastly, it is useful to underline that the meta-analysis addressing the risk of ND performed in moderate to severe OSA was based on six studies carried out in individuals with different clinical characteristics, such as members of the general population, normotensive elderly, and untreated and/or treated hypertensive patients. This clearly makes it inappropriate to extend our findings to specific populations.

### Limitations of the Study

Five different criteria were used to classify night-time and day-time periods in the studies included in the present meta-analysis. In addition, different definitions of abnormal nocturnal BP fall (systolic, systolic-diastolic, or mean BP) were adopted by the authors. These methodological differences in defining the ND pattern may have affected our findings. Classification of participants in D and ND, according to a 10% decrease of night-time BP as defined by a single ABPM recording does not accurately reflect a sustained BP trait, and its reproducibility over time is limited. Due to the cross-sectional design of the selected studies, a cause-effect relationship between the OSA and ND patterns remains unproven. We did not analyze the individual data from the original databases but only the results derived from revised papers. Mean BMI was significantly higher in subjects with OSA than in controls in three out of the seven studies included in the meta-analysis [19,26,27]. This could be considered a potential confounding factor. Moreover, the definition of hypopnea differed between the various studies in terms of percentage of air-flow reduction, inclusion or not of arousals, and oxygen desaturation. The use of different criteria in the definition of hypopnea can be a limitation in comparing the results of the selected studies and, consequently, of their meta-analysis. This also applies to the different methods of diagnosing OSA (polysomnography vs. polygraphy). Lastly, the restriction to papers published in English due to problems in interpreting reports in different languages may have partly influenced our findings.

## 5. Conclusions

Current evidence on the link between OSA and the ND pattern has been based, so far, on the results provided by single studies. Our meta-analysis focusing on a large pooled population of patients with OSA adds further information in this field by showing that the syndrome is associated with a significant increased risk of abnormal reduction in night-time BP. In a clinical perspective, this observation supports the view that the ND pattern may be regarded as a marker of OSA, especially when associated with clinical risk factors for sleep disordered breathing and, conversely, it supports the view that patients with OSA should be subjected to ABPM in order to identify and treat nocturnal hypertension.

## Figures and Tables

**Table 1:** Summary of the studies addressing the relationship between obstructive sleep apnea (OSA) and the non-dipping pattern (ND).

Author (Reference) Year Publication	OSA Sample Size (*n*)	Controls (*n*)	Men (*n*)	Mean Age (Years)	BMI (kg/m^2^)	Mean Night-Time BP (mmHg)	Setting	OSA Definition
Loredo [15], 2001	44	No	35	48	32	-	Uncomplicated NT and HTN	AHI ≥ 15
Tsioufis [16], 2008	62	70	56	48	32	123/78	Uncomplicated, untreated HTN	AHI ≥ 5
Sasaki [17], 2012	215	No	190	-	-	-	NT, untreated/treated HT	AHI ≥ 5
Onen [18], 2012	30	15	21	77	27	132/73	Elderly NT	AHI ≥ 15
Ishikawa [19], 2012	69	52	61	55	27	-	NT and HTN	AHI ≥ 15
Lee [20], 2014	63	640	36	60	24	-	General population	AHI ≥ 15
Seif [21], 2014	298	No	294	63	34	116/64	Cardiovascular disease	AHI ≥ 15
Sarinc Ulasli [22], 2014	62	No	43	52	32	115/73	Healthy NT	AHI ≥ 5
Sasaki [23], 2014	251	No	227	60	26	123/79	Prevalent HTN	AHI ≥ 5
Correa [24], 2017	26	55	12	44	34	115/70	Obese NT	AHI ≥ 15
Ma [25], 2017	56	No	40	48	27	-	Untreated HTN	AHI ≥ 5
Jenner [26], 2017	52	43	28	59	32	124/72	Treated HTN	AHI ≥ 15
Genta Pereira [27], 2018	77	76	33	64	30	129/70	Treated HTN	AHI ≥ 15
Sesizuka [28], 2018	264	-	219	55	28	123/81	Severe OSA	AHI ≥ 30

AHI = apnea/hypo-apnea index. BMI = body mass index. BP = blood pressure. HTN = hypertension. NT = normotension. OSA = obstructive sleep apnea. Data are presented as absolute numbers, mean ± SD, or inter-quartile range.

## Supplementary Materials

The following are available online at https://www.mdpi.com/2077-0383/8/9/1367/s1, Table S1: Data on definition of dipping pattern, night-time period, interval of night-time blood pressure measurements and type of ambulatory blood pressure monitoring devices provided by the selected 14 studies.
