# Supplementary material for: Blood Pressure Non-Dipping and Obstructive Sleep Apnea Syndrome: A Meta-Analysis
Source: J Clin Med. 2019 Sep 2;8(9):1367. doi: 10.3390/jcm8091367 (PMC6780266; doi:10.3390/jcm8091367)
Supplement: Supplementary file 1 [file jcm-08-01367-s001.pdf]

Supplementary Table S1. Data on definition of dipping pattern, night-time period, interval of night-time blood pressure measurements and type of ambulatory blood pressure monitoring devices provided by the selected 14 studies.

Author (reference) Year publication	ABPM Device	Definition of Dipping	Definition Night-time period	Interval of night-time BP measurements
**Loredo [15] 2001**	Spacelabs 90207	MAP decrease > 10%	10 PM- 6 AM	30 min
**Tsioufis [16] 2008**	Spacelabs 90207	SBP/DBP decrease >10%	12 PM-6 AM	30 min
**Sasaki [17] 2012**	FM 800 or FB 250*	MAP decrease > 10%	10 PM- 6 AM	30 min
**Onen [18] 2012**	Spacelabs 90207	MAP decrease > 10%	10 PM-7 AM	30 min
**Ishikawa [19] 2012**	TM 2425	SBP/DBP decrease >10%	Diary	30 min
**Lee [20] 2014**	Mobil-O-Graph	SBP decrease >10%	11 PM- 6AM	30 min
**Seif [21] 2014**	Spacelabs 90207	MAP decrease > 10%	10 PM- 6AM	30 mim
**Sarinc Ulasli [22] 2014**	Mobil-O-Graph	BP decrease > 10%	n. a.	30 min
**Sasaki [23] 2014**	FM 800 or FB 250	BP decrease > 10%	Diary	n.a
**Correa [24] 2017**	Spacelabs 90207	MAP decrease > 10%	Diary	30 min
**Ma [25] 2017**	Contec, CM 506 C	n.a.	10 PM -6 AM	60 min
**Jenner [26] 2017**	Spacelabs 90207	n-a.	Diary	20 min
**Genta Pereira [27] 2018**	Spacelabs 90207	SBP or DBP decrease > 10%	Diary	20 min
**Sesizuka [28] 2018**	FM 800	SBP decrease >10%	Diary	30 min

ABPM=Ambulatory blood pressure monitoring; MAP=mean arterial pressure; SBP=systolic blood pressure; DBP=diastolic blood pressure.
